# The route of infection determines *Wolbachia* antibacterial protection in *Drosophila*

**DOI:** 10.1098/rspb.2017.0809

**Published:** 2017-06-07

**Authors:** Vanika Gupta, Radhakrishnan B. Vasanthakrishnan, Jonathon Siva-Jothy, Katy M. Monteith, Sam P. Brown, Pedro F. Vale

**Affiliations:** 1 Institute of Evolutionary Biology, School of Biological Sciences, University of Edinburgh, Edinburgh EH9 3FL, UK; 2 Centre for Immunity, Infection and Evolution, University of Edinburgh, Edinburgh EH9 3FL, UK; 3 IGDR-CNRS UMR 6290, 2, Avenue Du Professeur Léon Bernard, 35043 Rennes, France; 4 School of Biology, Georgia Institute of Technology, Atlanta, GA 30332-0230, USA

**Keywords:** *Wolbachia*, invertebrate immunity, *Drosophila*, symbiont protection, infection tolerance, sexual dimorphism

## Abstract

Bacterial symbionts are widespread among metazoans and provide a range of beneficial functions. *Wolbachia*-mediated protection against viral infection has been extensively demonstrated in *Drosophila.* In mosquitoes that are artificially transinfected with *Drosophila melanogaster Wolbachia* (wMel), protection from both viral and bacterial infections has been demonstrated. However, no evidence for *Wolbachia*-mediated antibacterial protection has been demonstrated in *Drosophila* to date. Here, we show that the route of infection is key for *Wolbachia*-mediated antibacterial protection. *Drosophila melanogaster* carrying *Wolbachia* showed reduced mortality during enteric—but not systemic—infection with the opportunist pathogen *Pseudomonas aeruginosa*. *Wolbachia*-mediated protection was more pronounced in male flies and is associated with increased early expression of the antimicrobial peptide *Attacin A*, and also increased expression of a reactive oxygen species detoxification gene (*Gst D8*). These results highlight that the route of infection is important for symbiont-mediated protection from infection, that *Wolbachia* can protect hosts by eliciting a combination of resistance and disease tolerance mechanisms, and that these effects are sexually dimorphic. We discuss the importance of using ecologically relevant routes of infection to gain a better understanding of symbiont-mediated protection.

## 1. Introduction

Beneficial microbial infections are common throughout the animal kingdom, with profound effects on host physiology, behaviour, ecology and evolution [1–3]. Bacterial endosymbionts of insects, for example, are known to manipulate host reproduction [4,5], to alter the host's acquisition of essential nutrients [1,6] and to provide protection from the deleterious effects of parasites and pathogens [7,8]. *Wolbachia pipientis*—a maternally inherited, intracellular bacterium of arthropods and nematodes—is one of the best-studied microbial symbionts [9,10]. Its host range is vast, with recent estimates that 48–57% of all terrestrial arthropods [11], and at least 10% of all *Drosophila* species carry *Wolbachia* [12].

The ability of some *Wolbachia* strains to protect insect hosts from pathogenic infections makes it particularly relevant for potential bio-control of insect-vectored zoonotic infections, and more broadly relevant as modifiers of host ecology and mediators of pathogen-mediated selection in insects [9,10,13]. *Aedes aegypti* and *Aedes albopictus* mosquitoes, for example, have been shown to become more resistant to Dengue and Chikungunya viruses, as well as malaria-causing *Plasmodium* when they are experimentally transinfected with *Wolbachia* [14–16]. In *Drosophila*, there is also strong evidence that flies carrying *Wolbachia* are better able to survive infection by a number of RNA viruses [7,8].

In contrast with the strong evidence for *Wolbachia*-mediated protection from viral infections and being able to protect mosquitoes from bacterial challenge [16], its ability to protect its native fruit fly hosts from bacterial infections has not been clearly demonstrated [17,18]. In one study, *Wolbachia* did not affect the survival or immune activity of *Drosophila simulans* or *D. melanogaster* during systemic infection with *Pseudomonas aeruginosa*, *Serratia marcescens* or *Erwinia carotovora* [18], while another study found that the presence of *Wolbachia* had no effect on the ability to suppress pathogen growth during systemic infections by intracellular (*Listeria monocytogenes, Salmonella typhimurium*) or extracellular bacterial pathogens (*Providencia rettgeri*) [17]. Given that *Wolbachia* can provide broad-spectrum protection to mosquitoes against a range of pathogens, including bacteria [19], the lack of evidence for antibacterial protection in flies is puzzling.

Here, we show that the route of infection is key for *Wolbachia*-mediated protection in *Drosophila*, which we find to occur during enteric—but not systemic—infection by the opportunist pathogen *P. aeruginosa*. We exposed flies that were naturally infected with *Wolbachia*, and identical derived flies that were cured of *Wolbachia* infection, to *P. aeruginosa* either through intra-thoracic pricking (causing a systemic infection) or through the oral route of infection by feeding (causing an enteric infection). We monitored how within-host microbe loads and survival varied throughout the course of an enteric infection to assess if *Wolbachia*-mediated protection was due to differences in the bacterial clearance rate (resistance) or if it aided host survival in the presence of high microbe loads (tolerance); we also examined how these protective effects differed between male and female flies. We further characterized the expression of immune and damage repair genes previously shown to be involved in enteric bacterial infection in *Drosophila*.

## 2. Material and methods

### (a) Fly stocks

Experiments were carried out using long-term laboratory stocks of *D. melanogaster* Oregon R (OreR). This line was originally infected with *Wolbachia* strain wMel (OreR^Wol+^). To obtain a *Wolbachia*-free line of the same genetic background (OreR^Wol−^), OreR^Wol+^ flies were cured of *Wolbachia* by rearing them on cornmeal Lewis medium supplemented with 0.05 mg ml^−1^ tetracycline. This treatment was carried out at least 3 years before these experiments were conducted, and the *Wolbachia* status of both fly lines was verified using PCR with primers specific to *Wolbachia* surface protein (*wsp*): forward (5′–3′): GTCCAATAGCTGATGAAGAAAC; reverse (5′–3′): CTGCACCAATAGCGCTATAAA. Both lines were kept as long-term laboratory stocks on a standard diet of cornmeal Lewis medium, at a constant temperature of 18 ± 1°C with a 12 L : 12 D cycle. Prior to the experiment, fly lines were raised on Lewis food at 25°C, with a 12 L : 12 D cycle for at least two generations. To standardize the larval density of experimental flies, replicate vials were set up containing ten, 2- to 4-day-old mated females from each OreR^Wol−^ or OreR^Wol+^ fly line who were left to lay eggs for 48 h to ensure that larval densities were comparable across all replicates, and that offspring were age-matched (within 48 h). Maternal flies from each line were sampled from at least four different bottles in order to avoid potential confounding effects of bottle-specific differences in fly microbiota.

### (b) Bacterial cultures

*Pseudomonas aeruginosa* is a common Gram-negative bacterium with a broad host range, infecting insects, nematodes, plants and vertebrates, and is found in most environments [20,21]. Enteric infection of *Drosophila* by *P. aeruginosa* results in pathology to intestinal epithelia due to the formation of a bacterial biofilm in the crop, a food storage organ in the foregut [22,23]. In most enteric infections, *P. aeruginosa* growth is restricted to the crop, and is sufficient to cause death [22,24]. Infections were carried out using the *P. aeruginosa* reference strain PA14, which has been shown to have a very broad host range [25,26]. To obtain isogenic PA14 cultures, a frozen stock culture was streaked onto fresh LB agar plates and single colonies were inoculated into 50 ml LB broth and incubated overnight at 37°C with shaking at 150 r.p.m. Overnight cultures were diluted 1 : 100 into 500 ml fresh LB broth and incubated again at 37°C with shaking at 150 r.p.m. At the mid-log phase (OD_600_ = 1.0), we harvested the bacterial cells by centrifugation at 8000 r.p.m. for 10 min, washed the cells twice with 1×PBS and re-suspended the bacterial pellet in 5% sucrose. The final inoculum was adjusted to OD_600_ = 25, and this was the bacterial inoculum used for all flies inoculated orally (enteric infection).

### (c) Enteric and systemic bacterial infection

For systemic infection, flies were pricked in the pleural suture with a needle dipped in a mid-log phase (OD_600_ = 1.0) PA14 culture. Control flies were pricked with a needle dipped in sterile LB broth. For oral exposure (enteric infection), a concentrated PA14 inoculum (OD_600_ = 25) was spotted onto a sterile filter paper (80 µl per filter paper) and placed onto a drop of solidified 5% sugar agar inside the lid of a 7 ml Bijou tube. For the uninfected control treatment, filters received the equivalent volume of 5% sucrose solution only. Two- to 4-day-old flies were sex sorted and transferred individually to empty plastic vials: 180 (90 male and 90 female) OreR^Wol+^, and 180 (90 male and 90 female) OreR^Wol−^. Following 2–4 h of starvation, flies were transferred individually to 7 ml Bijou tubes, and covered with previously prepared lids containing a filter paper soaked in PA14 culture. Flies were left to feed on the bacterial culture for approximately 12 h at 25°C. Following this period, we sacrificed six exposed and two control flies and counted CFUs by plating the fly homogenate in *Pseudomonas* isolating media (PAIM). The remaining flies were transferred to vials containing 5% sugar agar and incubated at 25°C.

### (d) Survival assays

We carried out separate experiments to measure how the presence of *Wolbachia* affected fly mortality during either enteric or systemic infection, with identical fly rearing and bacterial cultural conditions as those described above. For each survival assay (enteric or systemic infection routes), 2- to 4-day-old flies were sexed and exposed in groups of 10 flies to PA14, as described above. For each combination of male or female OreR^Wol+^ and OreR^Wol−^ line, we set up 10 flies per 10 replicates vials. Flies that died from infection were recorded every hour until all flies had died (systemic infection), or every 24 h for up to 8 days (enteric infection).

### (e) Quantification of within-host bacterial loads in orally infected flies

Following the initial 12 h exposure, every 24 h, we randomly sampled five to seven live flies per sex and *Wolbachia* status and quantified the microbe loads present inside the flies. Briefly, a single fly was removed from the vial and transferred to 1.5 ml microcentrifuge tubes. To guarantee we were only quantifying CFUs present inside the fly, and not those possibly on its surface, each fly was surface sterilized by adding 75% ethanol for 30–60 s to kill the outer surface bacterial species. Ethanol was discarded and flies were washed twice with distilled water. Plating 100 µl of the second wash in LB agar confirmed this method was efficient in cleaning the surface of the fly (no viable CFUs were detected). Each washed whole fly was placed in 1 ml of 1× PBS in a 1.5 ml screw-top microcentrifuge tube, centrifuged at 5000 r.p.m. for 1 min and the supernatant was discarded. Two hundred microlitres of LB broth were then added to each tube and the flies were thoroughly homogenized using a motorized pestle for 1 min. A 100 µl aliquot of homogenate was taken for serial dilution and different dilutions were plated on PAIM agar plates, incubated at 37°C for 24–48 h and viable CFUs were counted.

### (f) Statistical analyses of host survival and microbe loads and tolerance

Fly survival was analysed using a Cox proportional hazards model to compare survival rates, with fly ‘sex’, ‘infection status’ and ‘*Wolbachia* status’ and their interactions as fixed effects. The significance of the effects was assessed using likelihood ratio tests following a *χ*^2^-distribution. For flies that were exposed orally to PA14, we compared between pairs of treatments (control versus infected or with and without *Wolbachia*) using the Cox risk ratios. In orally infected flies, changes in the bacterial load within-hosts were analysed with a linear model with log_10_CFU as the response variable, and fly ‘sex’, ‘*Wolbachia* status’ and ‘time (DPI)’ as a continuous covariate. To assess sex- and *Wolbachia*-mediated differences in how sick a fly gets for a given pathogen load (tolerance), for each time point, we took the survival probability (as a measure host health) and PA14 CFUs present within the flies (as a measure of microbe load) for five replicate flies in each sex/*Wolbachia* combination, and fitted a four-parameter logidsitic model to this relationship [27] (see the electronic supplementary material, table S1 and accompanying text for details). All analyses were conducted in JMP 12 (SAS). Full model output tables can be found in electronic supplementary material, tables S1–S7.

### (g) Gene expression

We tested for differences in the expression of genes known to be involved in either bacterial clearance (*PGRP-LC, PGRP-LE, attacin A*) or in the response to stress and gut damage (*gstD8, gadd45, CG32302*) during enteric bacterial infection [28–30] using qRT–PCR*.* Details on specific genes are given in the main text. Gene-specific primers are reported in the electronic supplementary material, table S2 and PCR conditions are reported in the electronic supplementary material.

## 3. Results

### (a) Flies carrying *Wolbachia* show reduced mortality during enteric but not systemic bacterial infection

**Figure 1.**
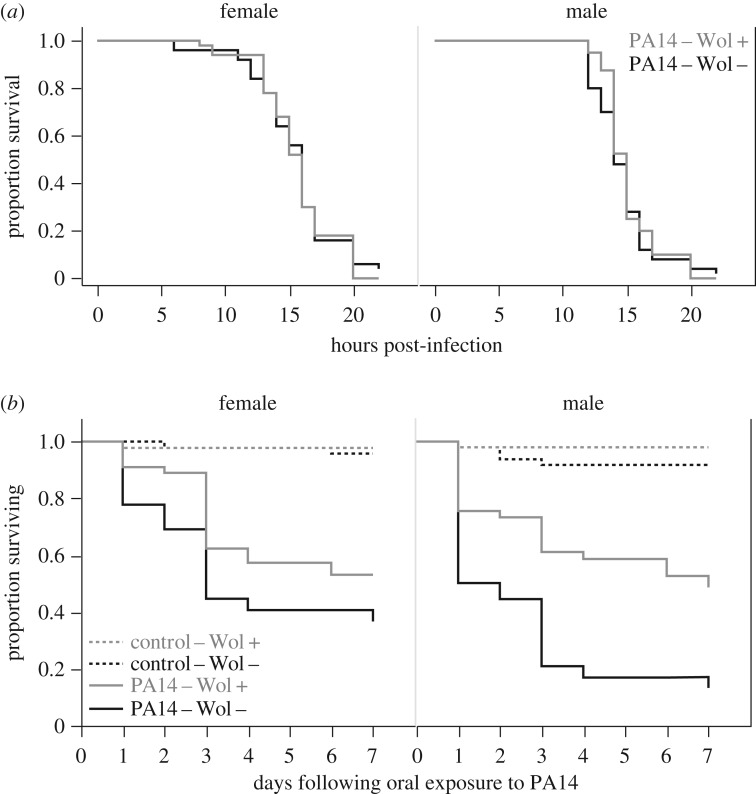
Fly survival after systemic oral infection with *P. aerugino*sa PA14. OreR^Wol−^ (black) and OreR^Wol+^ (grey) were either (*a*) pricked with a needle dipped in PA14 culture (OD = 1), or (*b*) left to feed on a PA14 culture (OD = 25) or on a control solution of 5% sugar for 12 h. Survival was monitored for 24 h (systemic infection) or daily (oral infection). In systemic infections, 100% of control flies survived over the 24 h period. In orally exposed flies, control flies are shown as dotted lines. Each data point shown is the mean of 10 replicate groups of 10 flies; these data were analysed using a Cox proportional hazard model.

All flies infected systemically with PA14 by intra-thoracic pricking died within 24 h (figure 1*a*), and in line with previous work [18], we did not detect any significant effect of *Wolbachia* status on the rate at which these flies died (Cox proportional hazard model, likelihood ratio *χ*^2^ = 0.003, d.f. = 1, *p* = 0.959), or any effect of sex (‘sex’ effect, *χ*^2^ = 0.860, d.f. = 1, *p* = 0.354); 100% of control flies (pricked with sterile LB broth) survived during the same period. Flies that ingested and acquired an enteric infection of PA14 died at a faster rate than control flies exposed only to a sucrose solution (figure 1*b*; ‘infection status’ effect, likelihood ratio *χ*^2^ = 64.27, d.f. = 1, *p* < 0.0001). Fly mortality during enteric infection was significantly affected by their *Wolbachia* status, but the extent of protection depended on fly sex (*Wolbachia* status × sex interaction *χ*^2^ = 8.50, d.f. = 1, *p* = 0.0036). This protective effect was not significant in female flies: the Cox risk ratio showed that females without *Wolbachia* were 1.58 more likely to die than infected females carrying *Wolbachia* (pairwise contrast: *p* = 0.06). The protection in male flies was more pronounced, as not carrying *Wolbachia* made PA14-infected males 2.26 times more likely to die than their infected *Wolbachia*-positive counterparts (pairwise contrast, *p* < 0.001; figure 1*b*).

### (b) The presence of *Wolbachia* affects initial bacterial clearance in males but not in females during enteric infection

**Figure 2.**
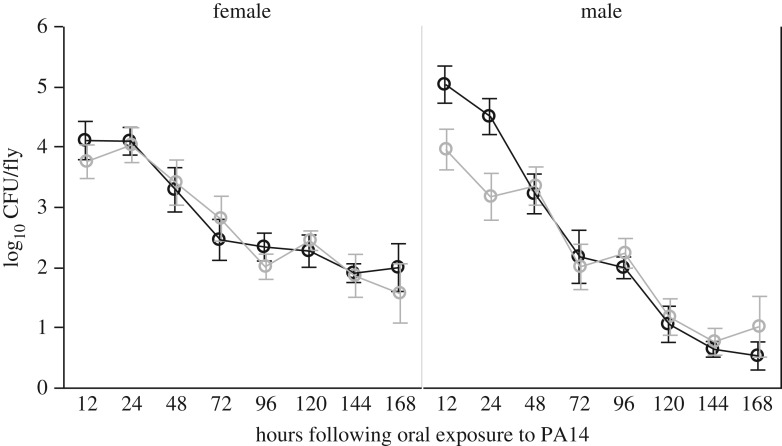
Within-host microbe loads. The number of viable within-host CFUs was quantified in five to seven individual live flies following 12 h of oral exposure, and then every 24 h for a week. Males and females are plotted separately for OreR^Wol−^ (black) and OreR^Wol+^ (grey) flies. Data shown are means ± s.e.m.

To understand the cause of the increased survival during enteric but not systemic infection protection, we focused on flies that acquired infection orally. Bacterial loads decreased over the course of the experiment in both male and female flies (figure 2) time effect (*F*_7,186_ = 48.81, *p* < 0.0001). We detected a significant statistical interaction between *Wolbachia* status, time and sex (electronic supplementary material, table S4), suggesting that the effects of *Wolbachia* on the rate of bacterial clearance are sex-specific. This was confirmed in a separate analysis for each sex: in females, there was no effect of *Wolbachia* on the rate at which PA14 was cleared (*Wolbachia* status × time interaction *F*_1,103_ = 0.032, *p* = 0.858), while in males, there was a significant effect of *Wolbachia* on how microbe loads changed with time (*Wolbachia* status × time interaction *F*_1,103_ = 9.28, *p* = 0.003). This effect is reflected in the difference in within-host CFUs measured at 12 and 24 h post-infection, where male flies harbouring *Wolbachia* showed 10-fold lower microbe loads compared with those without *Wolbachia* (figure 2; Wol+: 3.86 ± 0.22 log_10_ CFU; Wol−: 4.56 ± 0.22 log_10_ CFU; *F*_1,20_ = 5.27, *p* = 0.033). While we detected significant sex-specific effects of *Wolbachia* status on feeding (see the electronic supplementary material for feeding assay details and table S3 and figure S1), they were not consistent with changes in microbe loads, which were higher in *Wolbachia*-positive males.

### (c) The presence of *Wolbachia* changes the disease tolerance profile of male flies

Independently of *Wolbachia* status, we observed that males and females showed different patterns of bacterial clearance over time (figure 2; electronic supplementary material, table S4 ‘time × sex’ interaction). While males appeared to be able to clear the infection almost entirely within a week (mean ± s.e.m. 0.85 ± 0.29 log_10_ CFU per fly at 168 h post-exposure), females appeared to stop clearing infection after 96 h, maintaining a relatively stable bacterial load of about 100 CFUs per fly until the end of the experiment (figure 2). While we might expect this to result in higher female mortality, female flies showed similar survival to males following gut infection (figure 1*b*). Male flies, however, experienced increased survival when they were *Wolbachia-*positive compared with *Wolbachia*-negative males (figure 1*b*), even though the rate at which both groups clear infection appear identical (figure 2). This suggests that males benefit from increased infection tolerance in the presence of *Wolbachia*.

**Figure 3.**
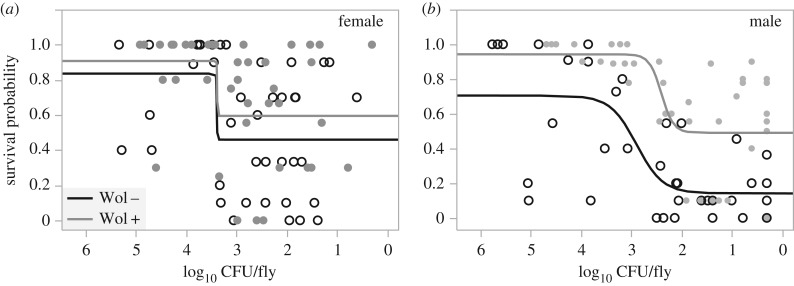
Disease tolerance. To measure disease tolerance, we analysed the relationship between host health and microbe loads. For each time point, we plot the survival probability (as a measure of health) against the microbe load (number of CFU per fly) for five biological replicates per sex and *Wolbachia* combination. Here, we show the fit of a four-parameter logistic model to the data (see electronic supplementary material, table S1 for model fits, and accompanying text for analysis details). The *x*-axis is reversed to read from beginning to the end of the infection (only clearance occurred).

To better assess these differences in disease tolerance, we analysed the relationship between host health and microbe load for matching time-points (see the electronic supplementary material for details on analysis of disease tolerance; figure 3). In all cases, a nonlinear four-parameter logistic model described these data better than a linear model (electronic supplementary material, table S1). In female flies, the logistic model explained one-quarter of the variance (*R*^2^ = 0.24), and a formal parallelism test found that the curves did not show significantly different shapes according to *Wolbachia* infection status (*F*_3,72_ = 0.886, *p* = 0.452). In male flies, the four-parameter logistic model explained over half the total variance (*R*^2^ = 0.57), and a formal parallelism test revealed significant differences in the shapes of these two tolerance curves between *Wolbachia*-positive and *Wolbachia*-negative males (*F*_3,72_ = 2.98, *p* = 0.037). These differences arise not only to the consistently lower maximum and baseline survival in *Wolbachia*-negative males regardless of microbe load (figure 3), but also due to differences in the inflection point of each curve which occurs later in the infectious period in *Wolbachia*-positive male flies (figure 3).

### (d) *Wolbachia*-positive flies show increased expression of immune deficiency pathway genes during the early stages of enteric infection

**Figure 4.**
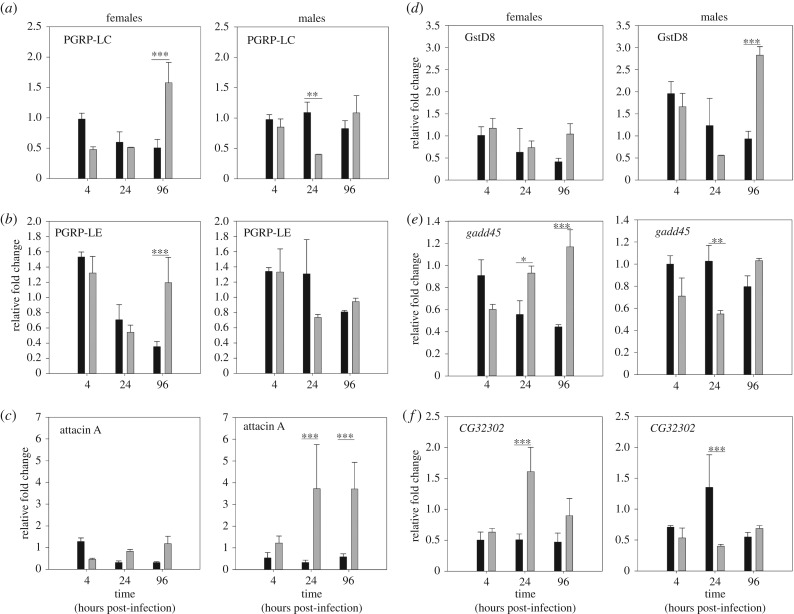
Gene expression relative to *rp49* control gene in infected flies relative to uninfected flies. The expression of genes involved in IMD-mediated antimicrobial immunity were measured: (*a*) PGRP-LC, a peptidoglycan pattern recognition molecule in the anterior fly midgut; (*b*) PGRP-LE, an intracellular peptidoglycan active in the posterior midgut; and (*c*) Attacin A, an AMP activated during infection by Gram-negative bacteria. We also measured the expression of GstD8—involved in ROS detoxification (*d*) and other genes involved in tissue damage repair (*gadd45*) (*e*) as well as and a component of the peritrophic matrix (*CG32302*) (*f*). *Wolbachia*-positive flies are shown in grey, and *Wolbachia*-negative flies in black. Data show the mean ± s.e. of pooled technical duplicates for three biological groups of five flies for each sex/*Wolbachia* combination, exposed orally to *P. aeruginosa* infection.

The immune deficiency (IMD) pathway is known to play an active role in the response to enteric bacterial infection [28,29]. We therefore tested whether flies carrying *Wolbachia* showed increased expression of genes involved in IMD-mediated antimicrobial immunity. Specifically, we measured the expression of genes that have been previously shown to be upregulated during enteric bacterial infection in *D. melanogaster* [28]: PGRP-LC, a peptidoglycan trans-synaptic signalling molecule that acts as a pattern recognition molecule in the anterior fly midgut [29]; PGRP-LE, an intracellular peptidoglycan that is especially active in the posterior midgut [29]; and Attacin A, an antimicrobial peptide (AMP) that is triggered by the IMD pathway during infection by Gram-negative bacteria [30]. In all genes, we detected significant time-dependent effects of *Wolbachia* status, and for the expression Attacin A, we also detected sex-dependent effects of *Wolbachia* carriage (see electronic supplementary material, table S6; figure 4); for these significant interactions, we report the relevant pairwise contrasts. In *Wolbachia*-positive females, we observed a significant increase in expression relative to uninfected females of PGRP-LC (figure 4*a*, *p* = 0.0002) and PGRP-LE (figure 4*b*, *p* = 0.004) at 96 h post-infection. Overall, there was no effect of *Wolbachia* on the expression of either receptor gene in male flies, but we observed a significant three- to fourfold increase in the expression of the AMP Attacin A in *Wolbachia*-positive males at both 24 h (*p* = 0.002) and 96 h (*p* < 0.001) post-infection (figure 4*c*).

### (e) *Wolbachia* is associated with higher expression of the reactive oxygen species detoxification gene *gstD8* in males during enteric infection

We hypothesized that in addition to the antimicrobial activity of Attacin A, mechanisms involved in detoxifying reactive oxygen species (ROS), commonly produced during enteric infection with PA14 [31], could also underlie the differences in survival between flies with and without *Wolbachia* (figure 1). The expression of GstD8—involved in ROS detoxification [31]—showed significant sex-specific effects of *Wolbachia* carriage over time during enteric infection with PA14 (electronic supplementary material, table S6). GstD8 expression was significantly higher at 96 h post-infection in males harbouring *Wolbachia* compared with those without the symbiont (figure 4*d*, *p* = 0.001), while no difference was observed in female GstD8 expression according to *Wolbachia* status (*p* = 0.08, figure 4*d*).

### (f) *Wolbachia* is associated with higher expression of epithelial repair genes in females during enteric infection

Oral infection often results in damage to insect guts [29], so we also measured the expression of genes involved in tissue damage repair (*gadd45*) and a component of the peritrophic matrix (*CG32302*) [28]. Both genes showed sex-specific effects of *Wolbachia* carriage that changed over time (electronic supplementary material, table S7). *Gadd45* expression was marginally higher in *Wolbachia*-positive females compared with those without *Wolbachia* at 96 h post-infection (figure 4*e*, *p* < 0.001). CG32302 expression was only transiently differentially expressed in *Wolbachia*-positive females at 24 h post-infection (figure 4*f*, *p* = 0.01), but not at the other time-points. *Wolbachia*-negative males showed a significantly higher expression relative to *Wolbachia*-positive males of both *gadd45* (figure 4*e*, *p* = 0.02) and CG32302 (figure 4*f*, *p* = 0.01) at 24 h post-infection, although this difference was no longer observed by 96 h post-infection.

## 4. Discussion

*Wolbachia* plays a key role in conferring protection from pathogens in their insect hosts [7,8]. In its natural host *Drosophila*, *Wolbachia*-mediated protection is especially evident during viral infections, but protection from bacterial pathogens in *Drosophila* had not been demonstrated to date. Here, we provide strong evidence that the route of infection is important for *Wolbachia*-mediated protection from bacterial infection. We find that *Wolbachia* can protect *Drosophila* from enteric bacterial infection by eliciting a combination of antimicrobial and damage repair mechanisms, and that these protective effects are sexually dimorphic.

### (a) The route of infection matters for *Wolbachia* protection

The role of *Wolbachia* in protecting hosts from infection, either by increasing resistance or tolerance, is known in *Drosophila*–virus interactions, but previous work testing for antibacterial protection in *Drosophila* did not find a significant effect of *Wolbachia* [17,18]. Typically, flies in previous studies were inoculated by intra-thoracic pricking or injection, and therefore experienced a systemic infection. In the wild, however, infections are more likely to be acquired through the faecal–oral route (during feeding on decomposing fruit), with most pathogens colonizing the gut before being shed through the faeces. *Drosophila–Wolbachia* interactions would therefore have co-evolved mainly under selection by pathogen infection in the gut, and any antibacterial protection that may have evolved as a consequence would not be expected to manifest during a highly virulent systemic infection [30,32]. Further, if *Wolbachia*-mediated protection is especially efficient in the fly gut, the damage caused by a generalized systemic infection could overwhelm any localized protection by *Wolbachia*, which could explain the lack of observed protection in previous studies of systemic bacterial infection in *Drosophila*.

Work in a number of insect host species, including flies [32,33], moths [34] and aphids [35], has highlighted how the route of infection can affect the progression and the outcome of disease due to differences in the mortality and the dynamics of pathogen growth. Distinct immune pathways are also elicited during systemic and enteric infection; recent work has shown that in *Drosophila*, the Toll-Dorsal pathway is required to defend from gut infection but not systemic infection by Drosophila C virus [36]. In addition to affecting the outcome of an infection at the individual level, these differences and immune deployment and disease outcome may even have more profound consequences for how hosts evolve in response to pathogens [32]. Studies of host resistance and tolerance should therefore favour natural routes of infection in order to gain a more realistic understanding of the mechanisms that hosts have evolved to fight infection.

### (b) *Wolbachia*-mediated protection is a combination of pathogen clearance and damage limitation

The mechanisms underlying *Wolbachia*-mediated protection are largely unclear, especially given that the extent of the protection and whether it acts to increase resistance or tolerance appear to be pathogen-specific [7,37,38]. In mosquitoes, *Wolbachia* protection appears to be involved in a combination of general immune priming [39], resource competition between *Wolbachia* and infectious agents [40], and the regulation of host genes involved in blocking pathogen replication [41]. However, mosquitoes have only been recently transinfected with *Wolbachia* and it is unclear if we might expect the same mechanisms to underlie protection in *Drosophila* which has a long coevolutionary history with *Wolbachia*. In *Drosophila*, *Wolbachia*-mediated antiviral protection is variable among strains of *Wolbachia* and correlates strongly with the reduction in viral titres within hosts [38], suggesting that *Wolbachia* generally enhances the ability to clear pathogens (increasing host resistance). These results contrast with work showing that *D. simulans* infected with *Wolbachia* strain wAu can withstand high virus titres without high levels of mortality [42], indicating that *Wolbachia* can, in some cases, also promote disease tolerance. Notably, *Drosophila–Wolbachia* associations that confer antiviral protection following systemic viral infection have also been found to protect adult flies following oral exposure to Drosophila C virus, although this was but not observed when flies were challenged as larvae [43].

Bacterial loads did not increase throughout the course of the infection, but were cleared at a near exponential rate (figure 2). Despite this, flies still died from infection, although the presence of *Wolbachia* was associated with a reduction in initial microbe loads and lower mortality in male flies, as well as an increase in the expression of the AMP Attacin A. One possibility is that most of the damage experienced by the host happens at the early stages of infection, as the greatest difference in male mortality happens within the first 48 h when bacterial loads are on average 10 times higher in *Wolbachia*-negative flies. It is therefore possible that the increased expression of *Attacin A* within the first 96 h post-infection (figure 4) may have led to the lower bacterial loads observed in the early stages of infection (figure 2), therefore minimizing gut damage caused by pathogen growth.

Given that we observed *Wolbachia*-associated changes in the tolerance profiles of male flies, we also chose to measure the expression of genes involved in damage repair. We investigated the expression of *gstD8*, involved in ROS detoxification, because it was previously shown to be upregulated during enteric infection in *Drosophila* with another bacterial pathogen, *E. carotovora* [28]. We found that the expression of *gstD8* was elevated in *Wolbachia*-positive males, but not female flies, following 96 h of oral exposure to *P. aeruginosa*, which is consistent with the increased survival observed in *Wolbachia*-positive males compared with males without the endosymbiont (figure 1*b*).

In addition to this detoxification response, we also measured the expression of genes involved in tissue damage repair (*gadd45*) and a component of the peritrophic matrix (*CG32302*). In males, the presence of *Wolbachia* did not result in an increase in these genes within 96 h of oral exposure to PA14, but females carrying the endosymbiont showed significantly higher expression than *Wolbachia*-negative flies of *gadd45*. This may indicate that *Wolbachia* could induce different damage limitation mechanisms in males and females. We also observed transient increases in the expression of CG32302, another component of gut renewal, in *Wolbachia*-positive females at 24 h post-infection. There was also a transient increase in expression at 24 h post-infection of *gadd45* and *CG32302* in *Wolbachia*-negative males (figure 4). We interpret these increases as a response to increased damage to gut tissue cause by the 10-fold higher bacterial loads in these flies after 24 h (figure 2), which was avoided in *Wolbachia*-positive males by *attacinA*-mediated clearance.

While previous work found no difference in genome-wide expression levels in adult *Drosophila* with or without *Wolbachia* [44], and only mild upregulation of immune genes has been reported in *Drosophila* cell lines that are transiently infected [45], our gene expression results indicate that *Wolbachia*-mediated protection from enteric bacterial infection relies on a combination of antimicrobial activity and damage repair mechanisms.

### (c) Sex differences in immunity and *Wolbachia*-mediated protection

A clear result from our work is that males and females vary in their ability to clear (figure 2) and tolerate infection (figure 3). While males and females are generally susceptible to the same pathogens, sexual dimorphism in the immune response is apparent in a wide range of species [46–48], and is documented for all classes of viral, bacterial, fungal and parasitic infections (see [49] for review). In invertebrate hosts, and especially in *Drosophila*, most studies investigating the ability to resist or tolerate bacterial and viral infections have focused primarily on the underlying immune mechanisms [21,29,50–52]. Typically, these studies have not focused on sexual differences in these mechanisms (but see [53]). However, our results, together with a large body of work on immune sexual dimorphism [54], show that resistance and tolerance mechanisms are likely to vary between males and females. The causes of sex differences in immunity are not clear, but one likely source of variation is that many immune genes are linked to sex chromosomes [55] and so X-linked regulators of fly innate immunity could underlie the sexually dimorphic clearance and tolerance response that we observed.

Moreover, this sexual dimorphism was modified by the presence of *Wolbachia*. We found that the tolerance curves of *Wolbachia*-positive males were always higher than those without *Wolbachia*, indicating that the presence of the endosymbiont results in greater health throughout the infection. However, we did not observe the same level of protection in female flies (figures 1–3). It is also notable that the inflection point of the curve (indicating a severe decline in survival) occurs much later in the infection in *Wolbachia*-positive males (although it does occur eventually), and that the overall severity of these infections in reduced (the baseline of the curve is higher) in *Wolbachia*-positive males.

This outcome was unexpected because maternally inherited symbionts, such as *Wolbachia*, are well known to use specific adaptive strategies to spread and persist within insect populations, usually providing fitness benefits to female hosts. This makes the greater protection in males surprising. One possibility is that the level of protection we observe in females is in fact the best adaptive strategy for *Wolbachia*, especially if the mechanism of protection (an increase in the expression of AMPs in males) could also result in lower *Wolbachia* titres, and hence lower *Wolbachia* fitness. Therefore, a possible explanation for lower antibacterial protection in females is that *Wolbachia* evolution has resulted in a balance between the fitness benefits to *Wolbachia* of reduced host pathology against the fitness costs of reduced *Wolbachia* titre.

## 5. Concluding remarks

Together, our results show that *Wolbachia* can protect *Drosophila* from enteric bacterial infection by eliciting a combination of antimicrobial and disease tolerance mechanisms associated with an initial upregulation of antimicrobial activity, and that these protective effects are sexually dimorphic. Future studies of symbiont-mediated protection should therefore favour natural routes of infection in order to gain a more realistic picture of the mechanisms that hosts have evolved to fight infection.

## Supplementary Material

Supplementary material

## Data accessibility

All data presented in this manuscript can be accessed on the Dryad Data Depository doi:10.5061/dryad.d0vp8 [56].
