# Supplementary material for: The route of infection determines Wolbachia antibacterial protection in Drosophila
Source: Proc Biol Sci. 2017 Jun 7;284(1856):20170809. doi: 10.1098/rspb.2017.0809 (PMC5474083; doi:10.1098/rspb.2017.0809)
Supplement: Supplementary material [file rspb20170809supp1.pdf]

Electronic Supplementary Material (ESM)

The route of infection determines *Wolbachia* antibacterial protection in *Drosophila*

Vanika Gupta [1§], Radhakrishnan B. Vasanthakrishnan [1,3§], Jonathon Siva-Jothy[1], Katy M. Monteith[1], Sam P. Brown[4], Pedro F. Vale[1,2*],

Analysis of disease tolerance

Disease tolerance is defined as the ability to maintain health relative to changes in microbe loads during an infection[1–3]. It is possible to analyse tolerance as the time-ordered health trajectory of a host as microbe loads change [4–7]. To assess the form of the health/microbe relationship, we fit linear and non-linear 4-parameter logistic model separately to the time-matched survival/microbe load plots. In all cases, the 4 parameter logistic model – which is commonly used to asses dose-response curves [8] - outperformed the linear fit.

Table S1. Fits of non-linear tolerance curves (Figure 4)

Sex	Model	AICc	AICc Weight	R-Square
Female	Logistic 4P	59.833	0.806	0.240
	Linear	62.683	0.194	0.110
Male	Logistic 4P	18.979	0.511	0.573
	Linear	19.070	0.489	0.517

The 4-parameter logistic model is useful to compare how its maximum (reflecting health in the initial stages of infection), baseline (reflecting the lowest survival reached during the experiment), inflection point (the point at which fly survival reached halfway between the baseline and maximum), and the growth rate (reflecting the rate at which fly survival plummets) vary according to host sex and *Wolbachia* status. Each of these parameters may reflect distinct mechanisms of damage repair involved in host infection tolerance, so they are useful for further exploration of tolerance mechanisms [1,9]. To test if these tolerance curves differed with *Wolbachia* status, we tested the parallelism of the models by comparing the error-sums of square for a full model (where each group has different parameters in the logistic model) to a reduced model (where models share all parameters except the inflection point) [8].

Table S2. qPCR conditions and Primer list

Primer name	Gene function	Sequence (5'->3')
PGRP-LC Forward	IMD immune pathway-Extracellular receptor	TTGAACCAAAGTAAGATCAGAGAT
PGRP-LC Reverse		GTCCAGATATATTGTTGAATT
PGRP - LE Forward	IMD immune pathway-Intracellular receptor	GATGCCGACCAAAATACCAG
PGRP - LE Reverse		GTCTTCGAAATGTGTCGGAG
Attacin A Forward	IMD immune pathway-Antimicrobial peptide	GGCCCATGCCAATTTATTCA
Attacin A Reverse		CATTGCGCTGGAACTCGAA
Gst D8 Forward	Stress response	GGAATCCCGTGCCATTTTGA
Gst D8 Reverse		CCCATGTCGAAGTAGAGCCT
CG32302 Forward	Peritrophic matrix	CGATGGAGAACTGGAGGTGA
CG32302 Reverse		TATCAGTCACGCAGGTCAGG
Gadd45 Forward	Wounding stress	ACTGGACCTGGAGCTAGAGA
Gadd45 Reverse		CTTGGAGAGCACGTTGATGG
Wsp Forward	*Wolbachia* surface protein	CATTGGTGTTGGTGTTGGTG
Wsp Reverse		ACCGAAATAACGAGCTCCAG

Our aim was to test if the expression of these genes varied in a sex- or *Wolbachia*-specific manner in flies that were infected orally. Groups of 5 flies for each sex / *Wolbachia* combination were exposed orally to *P. aeruginosa* infection in triplicate, as described above, and then frozen in TRI reagent at 4, 24 and 96 hours post-infection. Total RNA was extracted from flies homogenised in Tri Reagent (Ambion), using a Direct-zol RNA miniprep kit, which includes a DNAse step (Zymo Research), reverse-transcribed with M-MLV reverse transcriptase (Promega) and random hexamer primers, and then diluted 1:10 with nuclease free water. The qRT-PCR was performed on an Applied Biosystems StepOnePlus system using Fast SYBR Green Master Mix (Applied Biosystems) with the following PCR cycle: 95°C for 2 min followed by 40 cycles of 95°C for 10 sec followed by 60°C for 30 sec. Three biological replicates and two qRT-PCR reactions per replicate (technical replicates) were carried out per experimental condition. Changes in gene expression were analysed relative to the expression of *rp49,* an internal control gene. The relative fold-change difference in expression between infected and health control flies was calculated as described in [10]. Briefly:

$$\text{Fold change} = 2^{-\Delta\Delta Ct}$$

Where, $\Delta\Delta Ct$ = [(Ct of Gene A – Ct of Internal control) of Infected sample] –

[(Ct of Gene A – Ct of Internal control) of Control sample]

The fold change difference obtained was analysed using 3-way ANOVA with sex (Male, Female), Time (4, 24 and 96 hours) and *Wolbachia* (Wol- and Wol+) as fixed factors.

Feeding assay

To test if differences in bacterial loads were due to differences in feeding rate, we measured the amount of food flies ingested over a 24-hour period. All flies used were 0-72-hour-old reared as described above. Flies were sex-sorted under CO_2 anaesthesia and starved for 2 hours in an empty vial before being individually placed into a vial containing Blue-dyed Lewis medium (Blue Dye number 1, 0.5g/litre). Between 22-24 individual flies per Sex/*Wolbachia* combination were set up. All flies were left to feed on the blue dyed medium for 24hours at 25 degrees under 12:12 light:dark cycle. After 24 hours, flies were frozen, and then decapitated to avoid inaccurate absorbance readings due to eye pigments. Each individual fly was then placed in an Eppendorf tube containing 100ul of ice-cold Ringer's solution, homogenised using a motorised pestle and centrifuged for 10mins at ~13300g at 20ºC. 80 µl of this blue-dyed supernatant was loaded into a 96-well plate and the absorbance was measured using a VersaMax microplate reader, recording absorbance at 520nm.

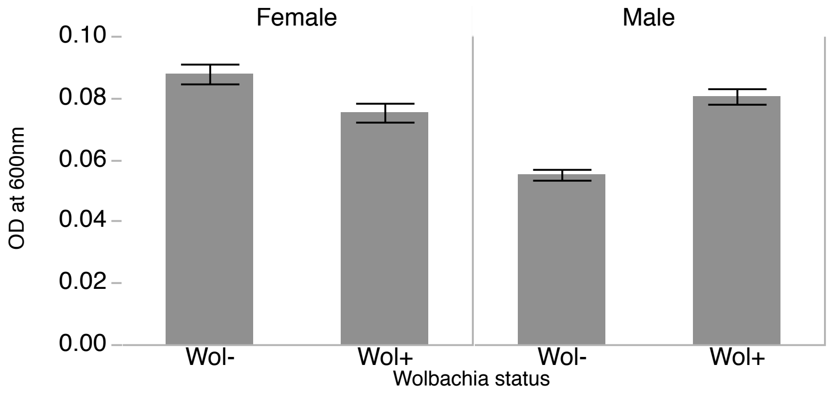

Figure S1. Feeding assay. Feeding was quantified by measuring the absorbance (at 600nm) of fly homogenate following 24h of feeding on blue-dyed Lewis medium (n=22-24 per treatment group).

Table S3: Linear model output for feeding amount (Figure 3a)

Source	DF	F Ratio	Prob > F
Wolbachia status	1	5.8116	0.0182
Sex	1	26.4530	0.0001
Wolbachia status*Sex	1	50.6306	0.0001

Table S5: Cox Proportional Hazards model for survival following oral infection (Figure 1B)

Source	DF	X^2	p-value
Sex	1	13.41	0.0003
Wolbachia status	1	27.71	0.0001
Wolbachia status*Sex	1	8.50	0.0036

Table S6: Linear model output for within-host CFUs (Figure 2)

**Source**	**DF**	**F Ratio**	**Prob > F**
Wolbachia status	1	0.5762	0.4487
Sex	1	20.2301	0.0001
time (hours)	1	325.7312	0.0001
Wolbachia status*Sex	1	0.2253	0.6355
Wolbachia status*time (hours)	1	4.5744	0.0337
Sex*time (hours)	1	22.1956	0.0001
Wolbachia status*Sex*time (hours)	1	5.6675	0.0182

Table S7: Statistical analysis of gene expression (Figure 5)

Gene	Effect	DF	F Ratio	p-value
pgrp - le	Sex	1	1.3667	0.2561
	Wolbachia	1	0.0008	0.9777
	Time	2	11.2416	0.0005
	Sex*Wolbachia	1	1.7106	0.2057
	Sex*Time	2	1.3765	0.2754
	Wolbachia*Time	2	4.835	0.0194
	Sex*Wolbachia*Time	2	1.5301	0.2408
pgrp - lc	Sex	1	0.8498	0.3676
	Wolbachia	1	0.0127	0.9113
	Time	2	3.5038	0.0496
	Sex*Wolbachia	1	2.7334	0.1139
	Sex*Time	2	0.8158	0.4565
	Wolbachia*Time	2	11.2636	0.0005
	Sex*Wolbachia*Time	2	3.4586	0.0513
att A	Sex	1	7.3169	0.0136
	Wolbachia	1	13.314	0.0016
	Time	2	1.0839	0.3573
	Sex*Wolbachia	1	9.7441	0.0054
	Sex*Time	2	1.9279	0.1715
	Wolbachia*Time	2	4.0477	0.0334
	Sex*Wolbachia*Time	2	0.3	0.7441
gstD8	Sex	1	20.5356	0.0002
	Wolbachia	1	3.8823	0.0628
	Time	2	6.0991	0.0086
	Sex*Wolbachia	1	0.0009	0.9763
	Sex*Time	2	2.9422	0.0758
	Wolbachia*Time	2	10.4952	0.0008
	Sex*Wolbachia*Time	2	4.4999	0.0243
gadd 45	Sex	1	1.7515	0.2006
	Wolbachia	1	0.4828	0.4952
	Time	2	0.7252	0.4965
	Sex*Wolbachia	1	12.1698	0.0023
	Sex*Time	2	0.0873	0.9168
	Wolbachia*Time	2	15.2386	0.0001
	Sex*Wolbachia*Time	2	3.8833	0.0376
cg32302	Sex	1	0.3484	0.5616
	Wolbachia	1	1.0641	0.3146
	Time	2	4.0685	0.0329
	Sex*Wolbachia	1	16.6289	0.0006
	Sex*Time	2	0.3844	0.6858
	Wolbachia*Time	2	0.7975	0.4643
	Sex*Wolbachia*Time	2	6.43	0.0069

Cited references

1. Ayres JS, Schneider DS. 2012 Tolerance of Infections. *Annu. Rev. Immunol.* **30**, 271–294. (doi:10.1146/annurev-immunol-020711-075030)

2. Medzhitov R, Schneider DS, Soares MP. 2012 Disease Tolerance as a Defense Strategy. *Science* **335**, 936–941. (doi:10.1126/science.1214935)

3. Råberg L, Graham AL, Read AF. 2009 Decomposing health: tolerance and resistance to parasites in animals. *Philos. Trans. R. Soc. Lond. B. Biol. Sci.* **364**, 37–49. (doi:10.1098/rstb.2008.0184)

4. Schneider DS. 2011 Tracing Personalized Health Curves during Infections. *PLoS Biol.* **9**, e1001158.

5. Doeschl-Wilson AB, Bishop SC, Kyriazakis I, Villanueva B. 2012 Novel methods for quantifying individual host response to infectious pathogens for genetic analyses. *Front. Livest. Genomics* **3**, 266. (doi:10.3389/fgene.2012.00266)

6. Lough G, Kyriazakis I, Bergmann S, Lengeling A, Doeschl-Wilson AB. 2015 Health trajectories reveal the dynamic contributions of host genetic resistance and tolerance to infection outcome. *Proc R Soc B* **282**, 20152151. (doi:10.1098/rspb.2015.2151)

7. Gupta V, Vale PF. 2017 Non-linear disease tolerance curves reveal distinct components of host responses to viral infection. *R. Soc. Open Sci.* , In press. (doi:10.1101/113217)

8. Gottschalk PG, Dunn JR. 2005 Measuring parallelism, linearity, and relative potency in bioassay and immunoassay data. *J. Biopharm. Stat.* **15**, 437–463. (doi:10.1081/BIP-200056532)

9. Vale PF, McNally L, Doeschl-Wilson A, King KC, Popat R, Domingo-Sananes MR, Allen JE, Soares MP, Kümmerli R. 2016 Beyond killing: can we find new ways to manage infection? *Evol. Med. Public Health* , eow012. (doi:10.1093/emph/eow012)

10. Livak KJ, Schmittgen TD. 2001 Analysis of relative gene expression data using real-time quantitative PCR and the 2(-Delta Delta C(T)) Method. *Methods San Diego Calif* **25**, 402–408. (doi:10.1006/meth.2001.1262)
